# Unveilling the hidden skillset: exploring non-technical skills in surgical education across spanish medical universities

**DOI:** 10.1186/s12909-024-05362-w

**Published:** 2024-04-05

**Authors:** Oves-Suarez B, García-Marín JA, Aguayo-Albasini JL, Soria-Aledo V

**Affiliations:** 1 School of Medicine, University of Murcia, Murcia, Spain; 2 Department of General and Digestive Surgery, University Hospital Morales Meseguer, Murcia, Spain; 3 Chief of Department of General and Digestive Surgery, University Hospital Morales Meseguer, Murcia, Spain

**Keywords:** Medical education, Surgery, Patient safety, Non-technical skills, Human factors

## Abstract

**Background:**

Non-Technical Skills (NTS) are cognitive, social, and personal resource skills that are crucial in complex and high-risk environments. The aims of our research are to determine the prevalence and content of NTS in the surgical rotation teaching guides of the Medicine Degree programs in Spanish Universities, to identify the most prevalent types and subtypes of NTS, and to analyze factors associated with the prevalence of surgical NTS in Medical Schools in Spain.

**Methods:**

Descriptive observational cross-sectional study involving the identification and collection of competencies outlined in the surgical rotation teaching guides of Spanish Medical Schools. Information regarding university performance was obtained from the Foundation for Knowledge and Development Ranking webpage. The “Non-Technical Skills for Surgeons” (NOTSS) system was used to classify each competency in the teaching guides as NTS (categories and elements) and technical skills. Disagreements were resolved through group consensus.

**Results:**

A total of 1,846 competencies were analyzed in surgical rotations of the Medicine Degree programs across 40 Spanish Universities, with 99 competencies identified as surgical NTS, accounting for 5% of the total. The most frequently identified surgical NTS were “Decision Making” (46%), “Communication & Teamwork” (25%), and “Leadership” (19%). Additionally, several NOTSS were not identified in any institution. Public universities and those including a greater number of competencies had a higher rate of surgical NTS competencies, and we did not find a correlation between surgical NTS competencies and quality indices of University Centers.

**Conclusions:**

There is a limited presence of surgical NTS in the educational plans of Spanish Universities.

## Background

Surgery stands as a pivotal and essential component of healthcare worldwide. Surgical safety is a global public health priority. In fact, 40–65% of events related to unsafe medical care occur within an operating theatre [1, 5].

The current challenges in surgery (an overall change of the medical model from paternalistic to cooperative, work more focused on the team and less individual, etc.) differ from those of the past, rendering technical skills and manual dexterity alone inadequate to ensure comprehensive quality care [1, 2]. Thus, the concept of Non-Technical Skills (NTS) has been introduced, encompassing cognitive, social, and personal resource skills crucial in complex and high-risk environments. In commercial aviation during the 1980s, once technological advancements mitigated common safety issues, it was realized that the “human factor” was the most frequent cause of aviation accidents. In 1981, United Airlines pilots became the first to receive training in key NTS under the “Crew Resource Management” (CRM) [3].

In 1997, James Reason, a psychologist from the University of Manchester, proposed the “Swiss cheese” model for risk analysis and management within organizations [4]. Presently, up to 60% of surgical adverse events result from NTS deficits [5–7]. NTS, categorized into “Situation Awareness,” “Communication & Teamwork,” “Decision Making,” and “Leadership,” are related to emotional intelligence and contribute to safe and efficient surgical performance [8, 9]. A behavioral error in the operating theatre can lead to a serious adverse event. According to Gawande AA et al. [7] and Vioque SM et al. [10], 43% of surgical errors result from communication failures. NTS, both at the individual and team levels, are interrelated and constitute an essential complement to technical skills.

Publications such as “Crisis Management in Anesthesiology” by Gaba D et al. [11], and projects like “MedTeams” [12] and “TeamSTEPPS” [13] developed in the United States, have adapted CRM programs to the field of Medicine. However, it wasn’t until 2004 when Fletcher G et al. introduced an innovative system called “The Anaesthetists Non-Technical Skills” (ANTS), the first behavior marker system for NTS training and assessment, specifically in anaesthesia [14]. Two years later, Yule S et al. created another NTS taxonomy and system named “Non-Technical Skills for Surgeons” (NOTSS) for surgeons [15]. In 2010, Mitchell L et al. developed “Scrub Practitioners’ List of Intraoperative Non-Technical Skills” (SPLINTS) for instrument nurse practitioners [16]. Presently, NOTSS [15] is one of the most evidence-based and validated systems for individual assessment, and “Oxford Non-Technical Skills” (NOTECHS) for team assessment [17].

Nevertheless, little effort has been made to enhance formal training in surgical NTS during undergraduate studies, despite the clear importance of knowledge and implementation for effective surgical team performance. Investment in research and educational innovation by Scientific Societies and Universities is greatly needed in the field of surgery to train competent professionals adapted to new technologies in an ever-changing surgical landscape (robotic surgery, surgical artificial intelligence, etc.). Considering that university education forms the cornerstone of medical training, focusing on the initial step of the surgical education pyramid, medical students, is crucial. Mastery of surgical NTS will allow future physicians to flourish both professionally and personally, as these skills are applicable across all domains of human knowledge.

Currently, there is limited scientific literature concerning the learning of surgical NTS, and in Spain, there is no research evaluating the prevalence, content, or implementation of surgical NTS among medical students. Furthermore, no validated system exists for the periodic assessment of the level of surgical NTS training achieved by future doctors. Hence, the objectives of our research are to determine the prevalence and content of NTS in the teaching guides of surgical rotations within the Medicine Degree programs at Spanish universities, identify the most prevalent types and subtypes of NTS, and analyze the factors related to the prevalence of surgical NTS within our country’s Medical Schools.

## Methods

Descriptive Cross-Sectional Observational Study through the identification and compilation of competencies outlined in the surgical rotation teaching guides of Medical Schools in Spain in the year 2022. The inclusion criteria were: Medical Schools located in Spanish territory with a curriculum that includes the subject “Surgical Rotation” and accessible teaching guides via web or email. Among the 49 Medical Schools in Spanish territory, teaching guides for surgery could be identified for 38 through their websites, while emails were sent to the remaining 11 secretariats, resulting in obtaining guides from only 2 institutions. The surgical rotation subject within the Medicine Degree program is included in all Medical Schools in Spain and provides students with the opportunity to apply theoretical concepts learned in the classroom in a real clinical setting. This helps them develop practical skills, gain experience in managing surgical patients, and explore various surgical specialties before making more informed decisions about their future medical careers.

Out of the 40 Medical Schools included in the study, 34 are public and 6 are private. Two categories of school size were considered, categorizing as small (< 200 incoming students) and large (≥ 200 incoming students). Information regarding university performance was obtained from the Foundation for Knowledge and Development Ranking (CYD) [18] website, collecting the following variables for all institutions: teaching and learning area (faculty qualifications, success rate, innovative teaching and assessment methods), research area (publications per faculty member, normalized impact of publications, highly cited publications), knowledge transfer area (private funds), international orientation area (foreign faculty, international publications, international research funds), regional development contribution area (regional publications, regional research funds), and employment rate area (Social Security affiliation rate after one year). High performance was understood as an indicator > 66th percentile, intermediate performance as 33rd ≤ indicator ≤ 66th percentile, and low performance as indicator < 33rd percentile.

The NOTSS [15] system was employed to classify each competency in the teaching guides as NTS (categories and elements) and technical skills (Table 1). Additionally, NOTSS elements were subdivided into three categories (1 item, 2 items, and ≥ 3 items NOTSS out of the total competencies, respectively). A database was designed in Excel (Microsoft) version 19.0 for data recording, where all collected data were archived, and identifying data of participating Medical Schools were protected and encrypted. Competencies were evaluated by a single evaluator who received training and guidance in NTS identification from experts in the NOTSS system at the Royal College of Surgeons of Edinburgh [15]. Disagreements were resolved through group consensus. An intraobserver reliability study was conducted in two periods of the study (first period in August 2022 and second period in October 2022). Intraobserver agreement analysis was assessed using Cohen’s weighted kappa test.

**Table 1:** NOTSS Taxonomy (categories and elements)

Categories	Elements
Situation awareness	Gather information
	Understand information
	Project and anticipate future state
Decision-making	Consider options
	Select and communicate options
	Implement and review options
Communication & Teamwork	Exchange information
	Establish a shared understanding
	Coordinate team activities
Leadership	Establish and maintain standards
	Support others
	Cope with pressure

Descriptive analysis presented qualitative variables through frequency distribution of category percentages, while quantitative variables were assessed for normal distribution using the Kolmogorov-Smirnov test, and indicators of central tendency (mean or median) and dispersion (standard deviation or percentiles) were provided. In bivariate analysis, the Pearson’s Chi-Square test was used for qualitative variables, Spearman’s rank correlation for quantitative variables, and Student’s t-test or one-way ANOVA for mean comparison. Statistically significant differences were considered when p-value was less than 0.05. Statistical analysis was conducted using the Statistical Package for Social Sciences (SPSS) program (IBM) version 19.0.

This research was carried out in accordance with the publication standards for observational studies outlined in the STROBE Statement [19].

## Results

Among the 40 reviewed Medical Schools, a total of 1,846 competencies were analyzed within the surgical rotation subjects, identifying 99 surgical NTS, which accounts for 5% of the total competencies. Based on the total competencies required as stipulated in the curriculum of the subject (average score/university = 47.5), NTS exhibited an average of 2.3 per university.

**Fig. 1:**
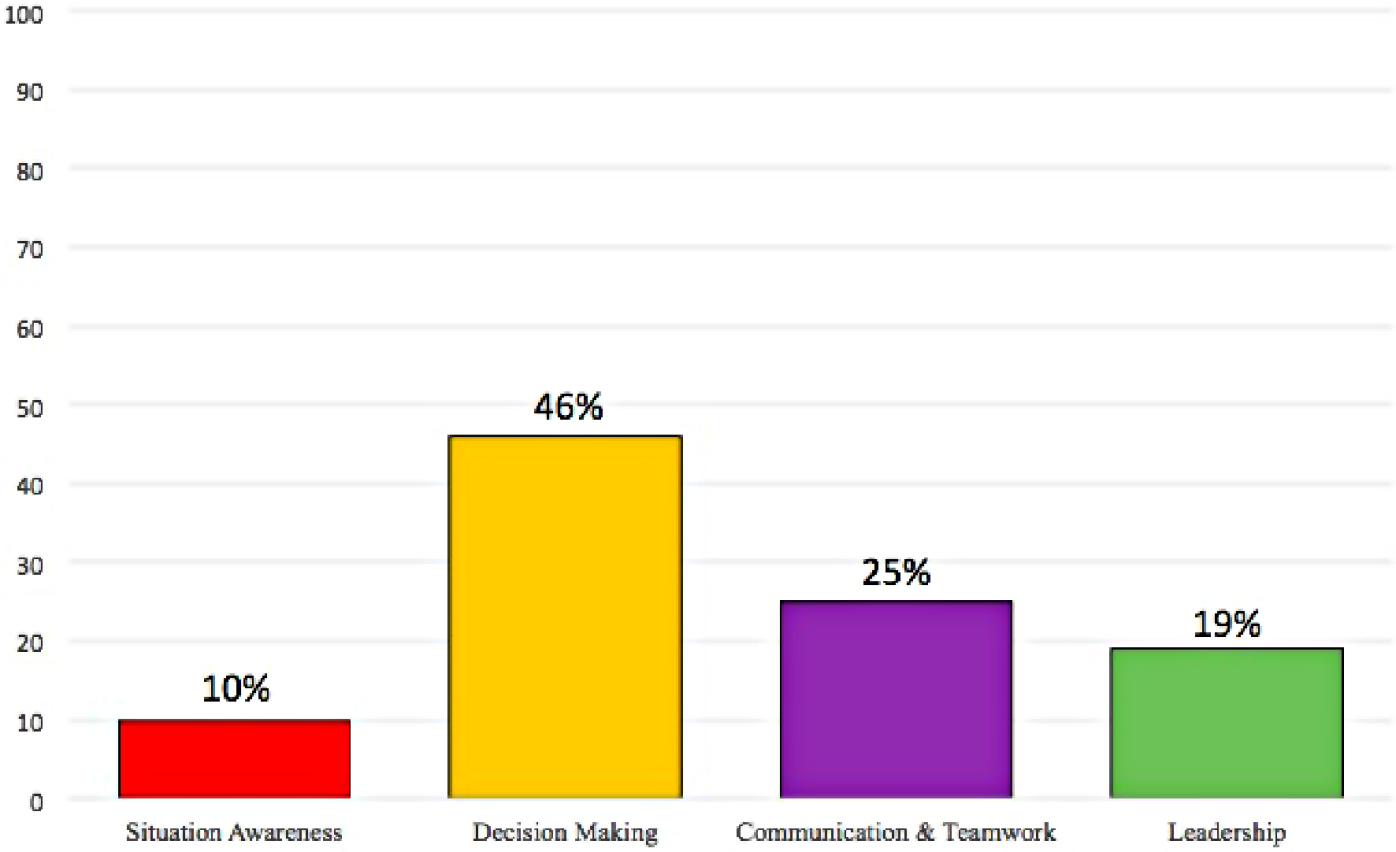
Distribution of non-technical skills in surgery in Spanish universities

The most frequent NOTSS categories were “Decision Making” (46%), “Communication & Teamwork” (25%), and “Leadership” (19%) (Fig. 1: Distribution of Non-Technical Skills in Surgery in Spanish Universities). Furthermore, several NOTSS were not identified in any institution. Table 2 outlines the prevalence of each NOTSS element.

**Table 2:** Prevalence of each element within its respective NOTSS category in surgical rotations of the medicine degree in Spanish universities

NOTSS Categories	Total Universities (*n* = 40)	1 item	2 items	≥ 3 items
Situation awareness	Recollect information	0 (0%)	6 (15%)	0 (0%)
	Understand information	0 (0%)	2 (5%)	0 (0%)
	Predict future state	0 (0%)	0 (0%)	0 (0%)
Decision-making	Consider options	14 (35%)	6 (15%)	1 (3%)
	Select and communicate options	4 (10%)	0 (0%)	0 (0%)
	Implement and review options	8 (20%)	2 (5%)	0 (0%)
Communication & Teamwork	Exchange information	4 (10%)	0 (0%)	0 (0%)
	Establish shared understanding	3 (8%)	1 (3%)	0 (0%)
	Coordinate team activities	5 (13%)	1 (3%)	3 (8%)
Leadership	Establish and maintain standards	5 (13%)	2 (5%)	2 (5%)
	Support others	3 (8%)	0 (0%)	0 (0%)
	Cope with pressure	0 (0%)	0 (0%)	0 (0%)

**Fig. 2:**
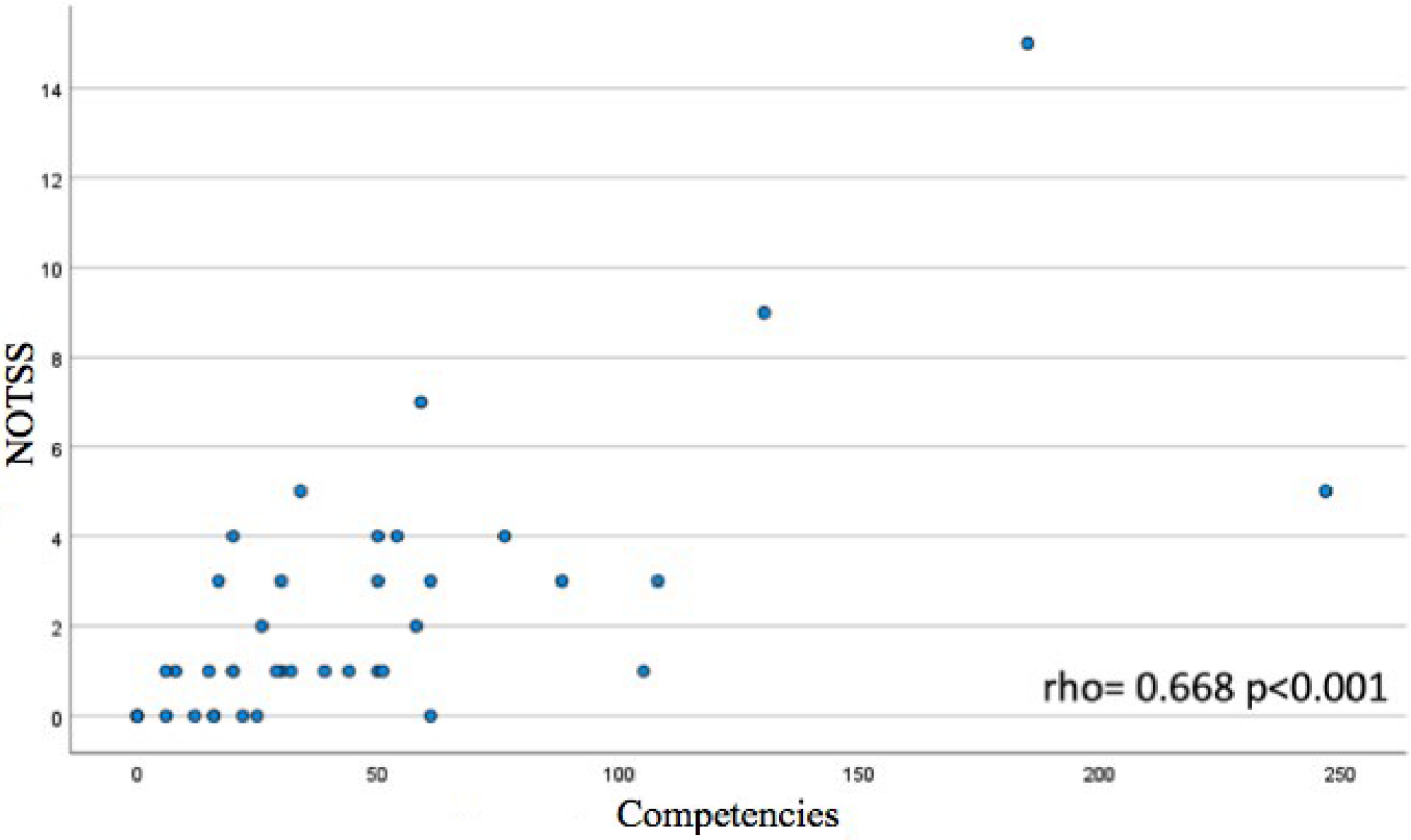
Correlation between total competencies and NOTSS identified in surgical rotations of the degree in medicine from Spanish Universities

Universities with a higher number of competencies in surgical subjects displayed more NTS in those subjects, showing statistical significance (rho = 0.668, *p* < 0.001) (Fig. 2: Correlation between total competencies and NOTSS identified in surgical rotations of the Degree in Medicine from Spanish Universities). No significant relationships were found between surgical NTS competencies and other performance indices of the University Centers (Table 3).

**Table 3:** Factors related to the prevalence of NOTSS in surgical rotations of the medicine degree in Spanish universities

	Total NOTSS (*n* = 99)	p^a^		Total NOTSS (*n* = 99)	p^a^
**University type** Public (*n* = 34) Private (*n* = 6)	2,6 ± 3 0,8 ± 1	0,002	**Private Funding**: High perfornance (*n* = 11) Intermediate performance (*n* = 12) Low performance (*n* = 11)	1,2 ± 1 1,8 ± 2 3,2 ± 3	0,133
**University size** ^**b**^ Small (*n* = 27) Large (*n* = 13)	1,9 ± 2 3,2 ± 4	0,082	**Foreingn Faculty** High performance (*n* = 12) Intermediate performance (*n* = 19) Low performance (*n* = 2)	2,5 ± 4 2,4 ± 2 3,5 ± 5	0,794
**Faculty Qualification** High performance (*n* = 11) Intermediate performance (*n* = 13) Low performance (*n* = 13)	2,8 ± 4 2,7 ± 3 1,5 ± 2	0,607	**International Publications** High performance (*n* = 14) Intermediate performance (*n* = 14) Low performance (*n* = 10)	2,5 ± 4 2,2 ± 3 2,3 ± 2	0,969
**Innovative teaching methods** High performance (*n* = 8) Intermediate performance (*n* = 6) Low performance (*n* = 8)	2,1 ± 2 2,7 ± 3 1,1 ± 1	0,569	**International Research Funding** High performance (*n* = 13) Intermediate performance (*n* = 16) Low performance (*n* = 4)	2,0 ± 3 1,7 ± 1 3,3 ± 3	0,244
**Publications per faculty member** High performance (*n* = 14) Intermediate performance (*n* = 12) Low performance (*n* = 11)	2,6 ± 4 2,4 ± 3 1,8 ± 2	0,873	**Regional Publications** High performance (*n* = 10) Intermediate performance (*n* = 14) Low performance (*n* = 14)	1,3 ± 2 2,5 ± 2 2,9 ± 4	0,415
**Normalized Impact of Publications** High performance (*n* = 10) Intermediate performance (*n* = 14) Low performance (*n* = 14)	1,7 ± 2 1,5 ± 1 3,6 ± 4	0,117	**Regional Research Funding** High performance (*n* = 12) Intermediate performance (*n* = 12) Low performance (*n* = 9)	2,2 ± 2 2,0 ± 3 1,8 ± 2	0,342
**Highly Cited Publications**: High performance (*n* = 10) Intermediate performance (*n* = 14) Low performance (*n* = 14)	1,4 ± 2 2,6 ± 4 2,7 ± 3	0,517	**Affiliation Rate with Social Security after One Year** High performance (*n* = 3) Intermediate performance (*n* = 2) Low performance (*n* = 4)	1,3 ± 2 3.5 ± 5 0,5 ± 1	0,529

NOTSS: Non-Technical Skills for Surgeons

Quantitative values expressed as mean ± standard deviation. Student’s t-test or one-way ANOVA, as appropriate

Medicine degrees offered by public Universities exhibited an average of 2.6 ± 3 surgical NTS competencies, whereas Medicine degrees offered by private Universities had an average of 0.8 ± 1, with the differences between these types of institutions being statistically significant (*p* = 0.002).

Regarding the number of students, in terms of university size based on the number of available spots for enrolling in the Medicine Degree program at the institution, no significant differences were found (*p* = 0.082). However, a slight trend was observed that Universities with ≥ 200 spots had a higher number of NOTSS in their curricula compared to universities with < 200 spots (3.2 ± 4 vs. 1.9 ± 2).

Regarding intraobserver agreement, the kappa index was 0.9 (95% CI 0.8-1.0).

## Discussion

The subject of “surgical rotation” within the Medicine Degree pertains to a practical training phase where medical students have the opportunity to rotate through different surgical specialties within a real clinical setting. The objective of this subject is to provide students with direct experience in the field of surgery, allowing them to acquire clinical skills and specific knowledge related to various surgical areas.

The outcomes of our study reveal the limited presence of surgical NTS within these subjects in Spanish Universities, figures that markedly differ from the total competencies that are evaluated to satisfactorily complete practical rotations in surgical specialties before graduation. Lee A et al. have affirmed this fact and underscored the necessity for NTS training during undergraduate studies within Medical Schools in Canada. Spanish Institutions exhibit a higher prevalence of NTS in their surgical rotations and differ in the order of the most prevalent NOTSS categories and elements identified compared to Canadian Institutions [20]. Since comprehensive figures on the prevalence of surgical NTS are not yet available, the trends in other countries remain unclear. What appears evident is that Medical students in both Spain and Canada are not receiving adequate training in surgical NTS, despite the evidence linking NTS to patient safety [21–23].

In our study, “Leadership” emerged as one of the least prevalent surgical NTS, with certain elements like “Coping with Pressure” not being identified in any institution. We believe that implementing specific leadership programmes for Medical students from the undergraduate level onwards could enhance the attitude of future professionals in critical situations.

The results of our study depict a greater prevalence of the NTS category “Communication & Teamwork” in surgical specialty rotations compared to other studies, despite communication failures being one of the foremost contributors to surgical errors today [7, 10, 24]. One possible explanation could be the challenge of imparting this skill to medical students.

Factors such as the type of university (public vs. private) have been associated with a higher number of NOTSS in the curricula of Spanish Medical Schools. This data has not been explored previously, and the rationale behind this observation is not straightforward to comprehend. While the observed differences in favour of public Institutions are statistically significant, it should be noted that the vast majority of Centers in Spain are public, and private Universities have a lesser tradition, which could explain these disparities; however, further insights from additional studies are warranted.

Surgical NTS, like any other skill, must be learned and can be honed through training [25–27]. Hence, an innovative teaching model is needed to enhance knowledge, interest, and bridge the “Learning Gap” of medical students concerning these matters. In this context, artificial intelligence or the metaverse could be novel and appealing educational tools for the youth due to their cutting-edge technology. Other strategies like case simulation, didactic courses, the GemaSim simulator, mentoring, or role-playing games have been extensively described in scientific literature as effective for acquiring NTS in risk-free environments [28–32].

One of the strengths of this study lies in the fact that the participating researchers possessed prior training in NTS and patient safety before designing the study. Additionally, the registration method assessed the technique and intraobserver variability in recording variables to ensure data reliability. Another strength of this research is that the team consisted of representation from medical students, senior surgeons, and teaching-research staff from the University, thus obtaining perspectives from all stakeholders involved in the medical training process.

Several methodological limitations in our study should be acknowledged. Firstly, the identification of competencies was carried out by a single observer; while we believe that observer training by experts and team resolution of doubts and conflicts have mitigated the potential impact of this limitation. Secondly, the NOTSS [15] system is not validated for application to medical students. However, we consider it a useful tool for the study’s objective. The third limitation stems from evaluating the competencies outlined in the teaching plan, but it doesn’t imply that teaching encompassing these behaviors and attitudes has not been imparted within theoretical or practical teachings of surgical subjects.

The most significant challenges in the future involve assessing the impact of implementing surgical NTS on patient safety-related outcomes and complications, and finally, securing the inclusion of surgical NTS training, refinement, and periodic evaluation as educational priorities by competent authorities and responsible bodies.

## Conclusions

Our study examined 1,846 competencies in surgical rotation subjects within the Medicine Degree across 40 Spanish Universities, identifying 99 competencies falling within surgical NTS, which constitutes 5% of the total. The most frequently identified surgical NTS include “Decision Making,” “Communication & Teamwork,” and “Leadership.” Public Universities and those with a higher number of competencies exhibit a higher rate of surgical NTS competencies, and no correlation has been found between surgical NTS competencies and the quality indices of University Centers.

## Data Availability

The datasets used and/or analysed during the current study are available from the corresponding author on reasonable request.

## Abbreviations

- NTS: - Non-Technical Skills
- CRM: - Crew Resource Management
- ANTS: - Anaesthetists Non-Technical Skills
- NOTSS: - Non-Technical Skills for Surgeons
- SPLINTS: - Scrub Practitioners’ List of Intraoperative Non-Technical Skills
- NOTECHS: - Oxford Non-Technical Skills
- SPSS: - Statistical Package for Social Sciences
